# Clinicopathological and prognostic value of lysyl oxidase expression in gastric cancer: a systematic review, meta-analysis and bioinformatic analysis

**DOI:** 10.1038/s41598-022-21402-1

**Published:** 2022-10-06

**Authors:** Zirui Jia, Jiacheng Gao, Yuhang Wang, Tingting Zhou, Xiangwen Zhang, Guo Zu

**Affiliations:** 1 Department of Gastrointestinal Surgery, The Dalian Municipal Central Hospital Affiliated to Dalian Medical University, No. 826 Southwest Road Shahekou District, Dalian, 116033 People’s Republic of China; 2 Department of Graduate School, Dalian Medical University, Dalian, China; 3 Department of Neurology, The First Affiliated Hospital of Dalian Medical University, Dalian, 116011 China

**Keywords:** Predictive markers, Prognostic markers

## Abstract

The association between the expression of Lysyl oxidase (LOX) and its clinicopathological parameters and prognosis in patients with gastric cancer (GC) is still disputed. We performed this meta-analysis and bioinformatics analysis to clarify the relationship between the expression and methylation level of LOX with its clinicopathological parameters and prognostic value. We applied odds ratios with a 95% confidence interval to study the associations between LOX expression and clinicopathological parameters and overall survival (OS) in GC patients. In addition, association analysis of promoter methylation levels and expression of LOX with its prognostic value was performed using the Cancer Genome Atlas (TCGA) and four Gene Expression Omnibus (GEO) datasets. The PRISMA 2020 checklist was used to guide the data extraction and analysis. This meta-analysis includes seven clinical studies with a total of 1435 GC patients. LOX expression was related to lymph node metastasis and tumor distant metastasis in GC patients, but not to gender, tumor differentiation, Lauren classification, or tumor depth of invasion. Patients with GC grouped in high-expression of LOX had a much worse OS than those in low-expression. In addition, TCGA and four GEO datasets with 1279 samples were included in the bioinformatics analysis. The bioinformatics analysis showed that patients with high LOX levels had poor OS; low levels of methylation at some cg sites in the LOX gene were strongly related to poor OS and PFS; and methylation levels of LOX are negatively correlated with advanced tumor stage. The conclusion from comprehensive DNA methylation and gene expression analysis supports LOX as a specific diagnostic and prognosis biomarker in GC. LOX expression was related to lymph node metastasis, tumor distant metastasis and poor prognosis in GC. Low methylation levels were related to advanced tumor stage and poor prognosis in GC. Integrative analysis supports LOX as a specific diagnostic and prognosis biomarker in GC.

## Introduction

Gastric cancer (GC) is one of the most commonly occurring cancerous tumors of the gastrointestinal tract, ranking fifth in incidence rate and fourth in terms of death among cancers^1^. Each year, over 1 million new cases are diagnosed, and roughly 783,000 people die^2,3^. GC has become a major health burden throughout the world. Despite notable improvements in the early detection, staging system, and treatment of GC, the five-year overall survival (OS) rate is still hovering around 40%^4,5^. In particular, patients with GC in stages II, III, and IV have a five-year survival rate of nearly 31%, 13%, and 3%, respectively^6^. The high mortality rate is mostly caused by a lack of cognition of cancer invasion and metastasis^7–9^. There is an urgent need to regard cancer progression as a continuous process that is dependent on intricate system of tumor microenvironment (TME)^10^. The continual evolution of the TME through the various clinical phases occurred in lockstep with cancer cell proliferation and invasion^11,12^. Therefore, to significantly reduce the high mortality and recurrence rates associated with GC, it is critical and urgent to find innovative biomarkers in TME for early diagnosis and prognosis.

The copper-dependent amine oxidase lysyl oxidase (LOX) is a secretory enzyme. Its primary role is to crosslink elastic and collagen fibers covalently and to remodel the extracellular matrix (ECM)^13^. LOX is important in building and mediating the TME milieu, via modulating the extracellular matrix (ECM) by posttranslational modification. Recent research found that LOX plays a critical role in tumor cell proliferation and survival^14^. According to a number of studies, LOX has been highlighted as a biomarker of tumor progression and metastasis in multiple cancers, such as colorectal, breast, and ovarian carcinomas, as well as bronchial cancers^15–18^. Recent studies suggested that LOX can be used as a marker for diagnosis and determining prognosis in GC patients^19^. However, clinical studies have described conflicting conclusions about the relationship between the expression of LOX and its clinicopathological parameters and prognostic value. Lai H et al. reported that the expression of LOX was not relevant to lymph node metastasis^20^. But Peng C et al. reported that LOX expression was positively associated with lymph node metastasis^21^. Jie He et al*.* reported that the expression of LOX was negatively associated with lymph node metastasis^22^. In addition, the researchers discovered that because of methylation and heterozygozity loss, the tumor suppressor gene of LOX is inactivated in GC and that the expression of LOX may be related to a better prognosis^23^. But several studies have shown that LOX expression is related to a poor prognosis^19,21^. Strong evidence-based clinical investigations and bioinformatic analyses are required to clarify the particular role of LOX in GC. Unfortunately, previous meta-analysis and bioinformatic analyses have not noticed and addressed these great and valuable controversies. Thus, we apply meta-analysis to clarify the association and existing conflict in LOX expression with clinicopathological parameters and prognostic value by using clinical studies. We also perform bioinformatic analysis using TCGA and GEO databases to further demonstrate and explore the function of LOX.

## Methods

The protocol for this meta-analysis is registered with PROSPERO (CRD42022284572). The PRISMA 2020 checklist was used to guide the data extraction and analysis (Supplementary Material 1). We used the PRISMA (Preferred Reporting Items for Systematic Reviews and Meta-Analysis) and AMSTAR (Assessing the methodological quality of systematic reviews) Guidelines to perform this meta-analysis^24^.

### Search strategy

Two authors (Zirui Jia and Jiacheng Gao) performed a literature search, and if a difference emerged, it was resolved by a third author (Yuhang Wang). Pubmed, Ovid Embase, Web of Science, Cochrane Library, ClinicalTrials.gov, and Chinese databases (CNKI, WanFang, WeiPu and CBM) were searched for articles published between the creation of the database and February 30, 2022. There were no specified limitations, such as language or country. The search strategy was made up of three primary parts that were connected by AND: (I) Stomach Neoplasms (eg, Gastric Cancer, Stomach Neoplasm, Stomach Cancer), (II) LOX protein, human (eg, LOX, lysyl oxidase), (III) patients (eg, Patient, Clients, Client).

### Clinical study selection

The studies listed below were chosen for inclusion: (I) The entire text of article is accessible; (II) the relationship between LOX expression and clinicopathological parameters or prognosis in GC patients was explored; (III) Immunohistochemistry (IHC) was used to evaluate LOX protein expression. The degree of LOX expression must be clearly; (IV) Randomized controlled trials (RCTs), prospective or retrospective approaches, and all other quantitative research designs were considered.

The following articles were found to be ineligible for inclusion: (I) experiments on animals or cells; (II) the research was presented in the form of a review article, case report, letter, comment, or conference abstract; (III) studies that were repeated; (IV) the content of the study is irrelevant to the subject.

The quality of study was determined by the Newcastle–Ottawa Scale (NOS). The NOS scores of 6 or above denote exceptional excellence in quality scores and are included in meta-analysis.

### Clinical data extraction

Two authors (Zirui Jia and Jiacheng Gao) separately extracted results from every study that qualified: issuing time, first author, country, study period, gender, Lauren classification, tumor differentiation, depth of invasion, tumor metastasis, lymph node metastasis and OS in one, three, and five years. If there was a dispute, it was decided by a third author (Yuhang Wang).

### Extraction of TCGA and GEO Data

The Cancer Genome Atlas (TCGA) database includes gene expression databases as well as clinical and methylation data. All fragment per kilobase million mapped reads (FPKM) data, methylation450 profile and clinical data with GC from the TCGA database, which includes 375 cases of GC and 32 normal controls, were downloaded using UCSC Xena (https://xena.ucsc.edu/). In addition, we searched the Gene Expression Omnibus (GEO) database with the keywords “gastric cancer”, “survival” and “Homo sapiens”. The dataset was selected with a sample size of > 50. Finally, the data sets "GSE84437", "GSE62254", "GSE29272", and "GSE57303" with 433, 300, 126, and 70 samples were downloaded.

### The expression of LOX mRNA and methylation levels in GC patients and healthy controls

Perl software was used to extract the mRNA expressions of LOX from the HTSeq of GC. The differential expression of LOX in GC compared to healthy controls was estimated using the ggpubr package in R software. By using Perl software, the methylation levels of LOX were obtained from the methylation450 profile. Using the R software packages dplyr and ggpubr, the methylation levels of cg sites in the LOX DNA promoter regions were analyzed in GC.

### Survival analysis of methylation levels of cg sites and the mRNA expressions of LOX

The mRNA expression and methylation levels of LOX in the median were used to divide GC patients into high and low expression groups. Survival analysis between high and low groups was performed using the survminer and survival packages.

### Statistical analysis

R (v4.1.3) and RStudio (v2.0.433) were used for all analyses. For dichotomous variables including gender, Lauren classification, differentiation, depth of invasion, lymph node metastases, and tumor metastasis, the odds ratio (OR) and 95% confidence interval (CI) were adopted. The hazard ratio (HR) and 95% CI were used to compare time-related prognostic information such as OS. All statistical tests were two-sided, and statistical significance was determined by a *P* value of less than 0.05. Meta-analysis was performed using the “meta” package. The results of the study evaluation were shown in forest plots, and the funnel plot was used to visually assess publication bias. Each study was excluded sequentially for the sensitivity analysis (SA). To analyze the effects of removing each study, the pooled OR or HR of the remaining studies were used. *I*^2^ statistics were applied to analyze heterogeneity, while funnel plots were utilized to test publication bias. A fixed effect model was proposed for Meta-analysis when heterogeneity was less than 40%, according to the Cochrane Handbook^25^. Otherwise, a random effect model should be considered. The *I*^2^ statistic from the Q test quantifies the heterogeneity of studies. The funnel plot is used to check for publication bias.

The difference in survival between the high and low expression groups in GC was calculated using Kaplan–Meier analysis. Categorical variables were estimated using the Chi-squared test based on between-group differences. The Kruskal–Wallis test was used to analyse continuous variables. By using the R package "pROC", the ROC curve with time dependence was utilized to discover the prognostic implications by comparing the AUC^26^. In addition, for ROC verification and AUC data calculation, a tenfold cross-approach was used.

## Results

### Description of studies

**Figure 1:**
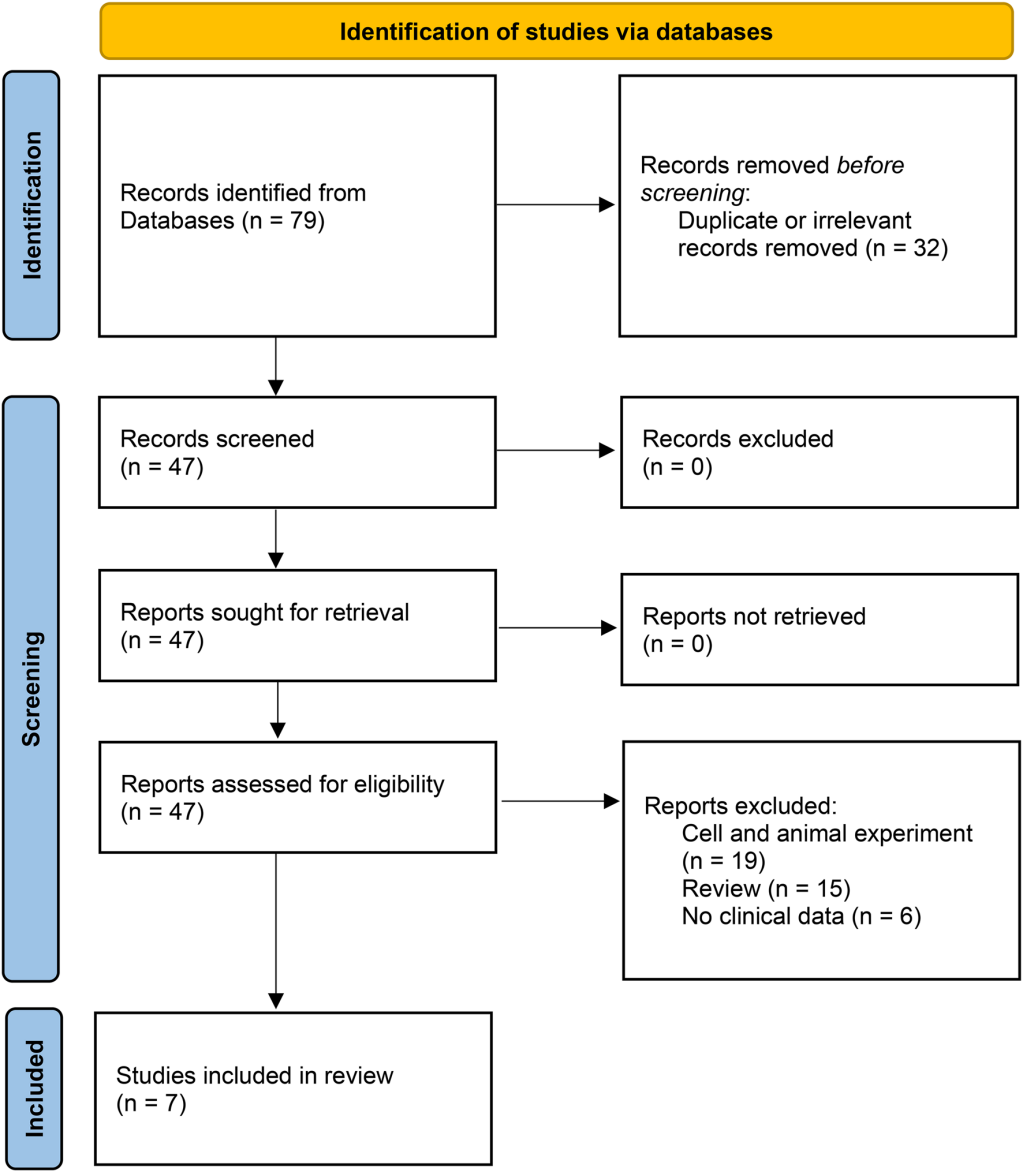
PRISMA 2020 flow diagram of literature selection.

From 9 database searches, we retrieved 79 articles (Fig. 1). 32 articles were found to be duplicates and were removed. 40 papers were eliminated from the remaining 47 for being unsuitable, including experiments on animals or cells (N = 19), review (N = 15), and no clinical data (N = 6). This meta-analysis included 1435 patients from seven studies, all of which matched the survey design^19–22,27–29^. The major collected data from included studies is summarized in Table 1 and Supplemental Material 2. Studies with a NOS score of 6 or above.

**Table 1:** Main characteristics of the eligible studies.

No	First author	Year	N	Gender (M/F)	During	Country	Method (H/L)	NOS
1	Han^19^	2019	140	112/28	2008–2012	China	IHC (H: 9–14, L: 0–9)	7
2	Peng^21^	2018	184	132/52	2002–2011	China	IHC (H:1–2, L:0–1)	8
3	Kasashima^27^	2015	544	312/232	2009–2015	Japan	IHC (H:5–8, L:0–5)	8
4	Lai^20^	2014	215	95/120	2009–2012	China	IHC (H:5–12, L:0–5)	7
5	Yalin Han^28^	2014	166	136/30	2007–2013	China	IHC (H:10–17, L:0–10)	7
6	Zhang^29^	2013	161	107/54	2002–2010	China	IHC (H:2–16, L:0–2)	7
7	He Jie^22^	2002	25	-	1999–2000	China	IHC (H:6–25, L:0–6)	6

*IHC* immunohistochemistry, *H* high expression, *L* low expression.

### Relationship between LOX expression and clinicopathological characteristics

**Figure 2:**
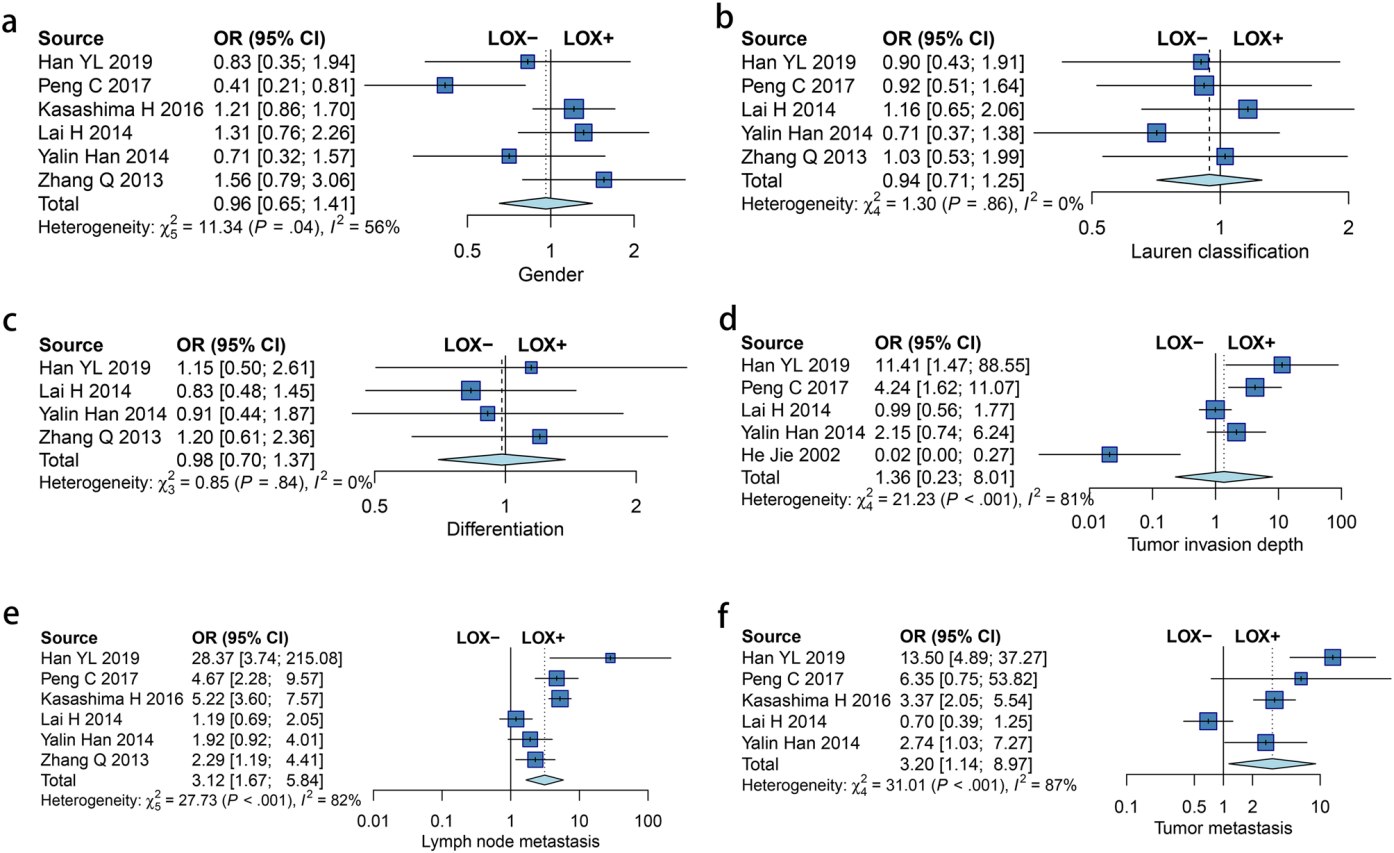
Forest plot for the relationship between LOX expression and clinicopathological characteristics: gender (**a**), Lauren classification (**b**), differentiation (**c**), depth of invasion (**d**), lymph node metastasis (**e**) and tumor distant metastasis (**f**).

The meta-analysis outcomes demonstrated that LOX expression was not related to gender (OR = 0.96, 95% CI = 0.652–1.415, *p* = 0.836), Lauren classification (OR = 0.943, 95% CI = 0.710–1.252, *p* = 0.686), differentiation (OR = 0.981, 95% CI = 0.702–1.373, *p* = 0.912), and depth of invasion (OR = 1.362, 95% CI = 0.232–8.013, *p* = 0.733) in patients with GC. Our results indicated that the expression of LOX was related to lymph node metastasis (OR = 3.12, 95% CI = 1.670–5.836, *p* < 0.001) and tumor distant metastasis (OR = 3.199, 95% CI = 1.141–8.972, *p* = 0.027) (Fig. 2).

**Figure 3:**
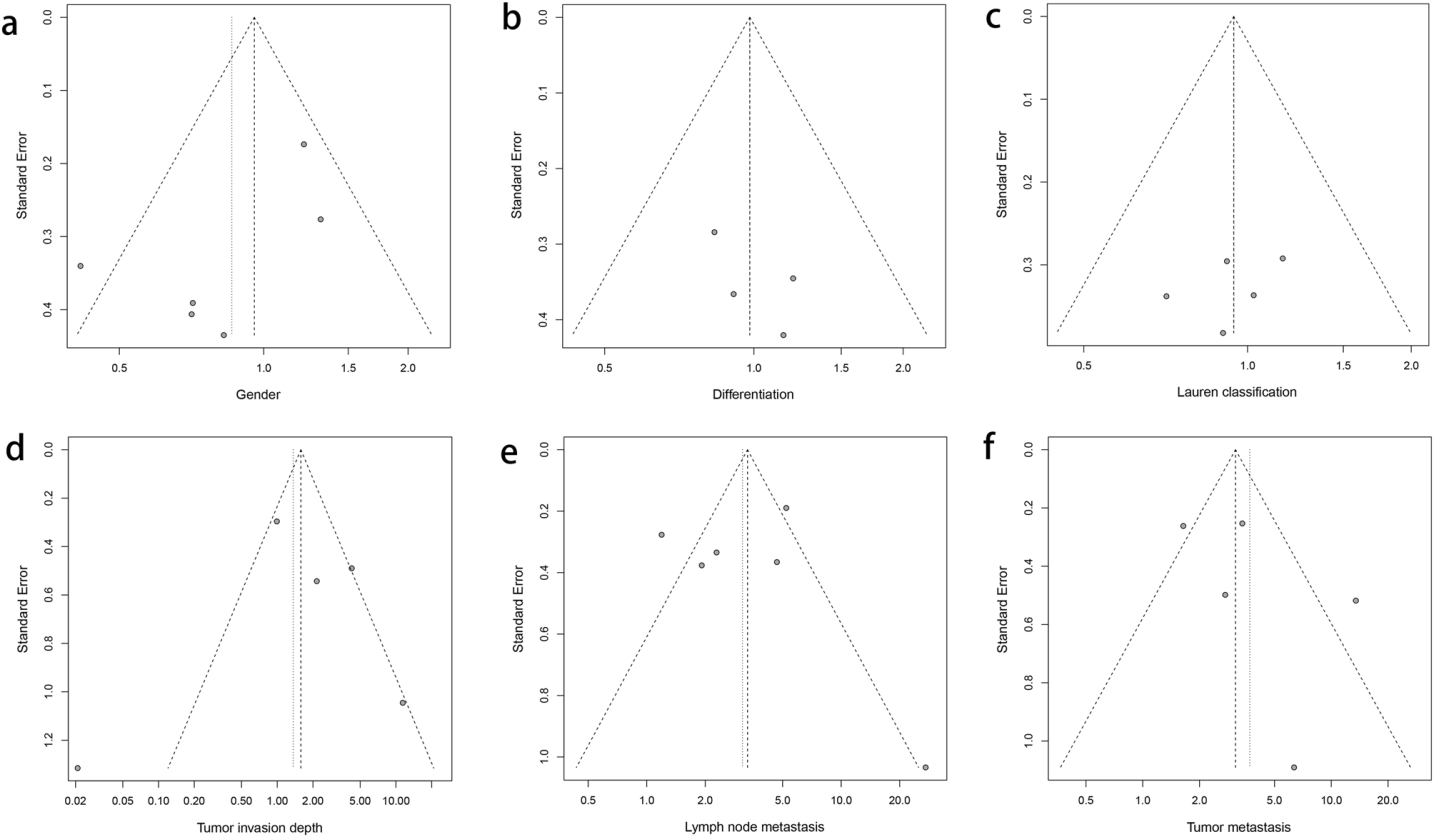
Funnel plot for the relationship between LOX expression and clinicopathological characteristics: gender (**a**), Lauren classification (**b**), differentiation (**c**), depth of invasion (**d**), lymph node metastasis (**e**) and tumor distant metastasis (**f**).

There was no significant publication bias found in gender, Lauren classification, differentiation, depth of tumor invasion, lymph node metastasis, or tumor distant metastasis since their p-values were larger than 0.05 in Egger’s test (*p* = 0.361, 0.503, 0.336, 0.635, 0.537 and 0.765, respectively) (Fig. 3).

### Association of LOX expression with OS

**Figure 4:**
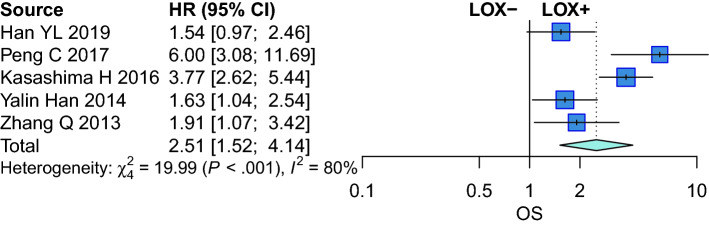
Forest plot for the association of LOX expression with OS.

Compared to GC patients with low LOX expression, the OS (HR = 2.509, 95% CI = 1.520–5.142, *p* < 0.001) were significantly poorer with high LOX expression in GC patients (Fig. 4).

**Figure 5:**
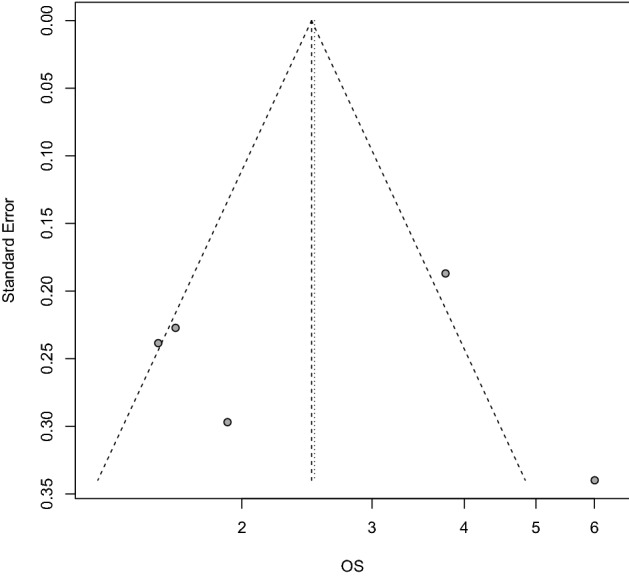
Funnel plot for the association of LOX expression with OS.

The funnel plot of OS was shown in Fig. 5. No significant publication bias was found in OS (Egger’s test, *p* = 0.951).

### Sensitivity analysis

Results of the SA revealed no changes in the significance or direction of OR in the relationship between LOX expression and gender, Lauren classification, differentiation, depth of invasion, lymph node metastasis, and tumor distant metastasis. SA also showed no changes in the significance or direction of HR in the relationship between LOX expression and OS. The forest plot of the SA is provided in the Supplementary Material 3.

### Prognosis and expression analysis of LOX in the TCGA and GEO databases

**Figure 6:**
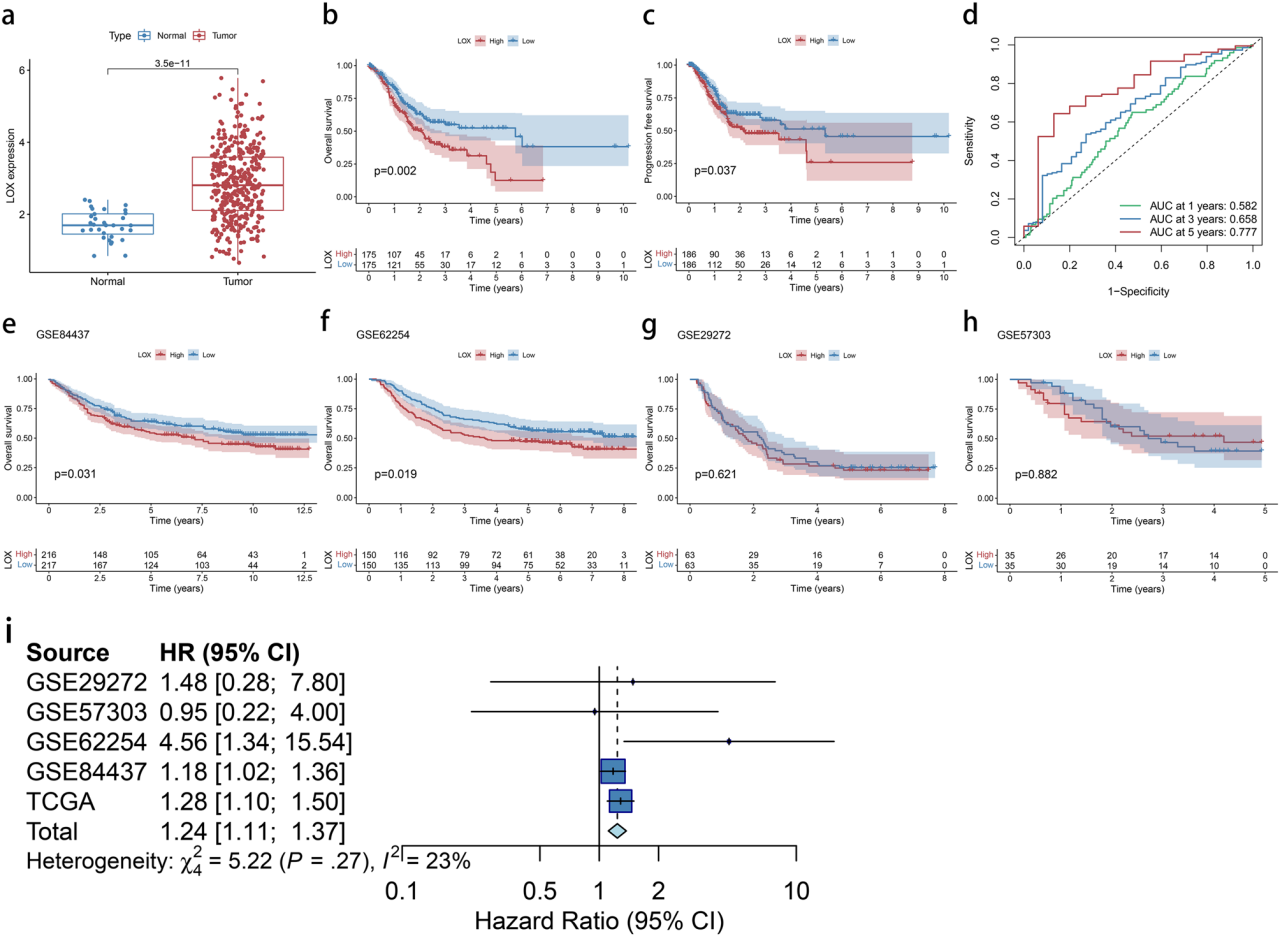
Prognosis and expression analysis of LOX in the TCGA and GEO databases. (**a**): The LOX gene was expressed at a significantly higher level in GC tissues than in normal (*P* < 0.001). (**b**): Patients with high LOX levels had poor OS than those patients with low (*P* = 0.002). (**c**): Patients with high LOX levels had poor PFS than those patients with low (*P* = 0.037). (**d**): Survival-reliant ROC curves confirm the prognosis value of LOX-based prognosis indexes. (**e**): K-M curves of the OS of 433 GC sufferers from GSE84437 (*P* = 0.031). (**f**): K-M curves of the OS of 300 GC sufferers from GSE62254 (*P* = 0.019). (**g**): K-M curves of the OS of 126 GC sufferers from GSE29272 (*P* = 0.621). (**h**): K-M curves of the OS of 70 GC sufferers from GSE57303 (*P* = 0.882). (**i**): Forest plot of high LOX expression versus low LOX expression (HR = 1.362, 95% CI = 1.11–1.37). Hazard ratios were analyzed with the fixed-effect model.

The LOX gene was expressed at a significantly higher level in GC tissues than in normal ones (*P* < 0.001) (Fig. 6a). Based on the median expression of LOX, we divided all GC patients into high and low groups to explore the prognostic significance of LOX expression in GC patients. Compared to patients with low LOX levels, those with high LOX levels had poor OS (*p* = 0.002, Fig. 6b and progression-free survival (*p* = 0.037, Fig. 6c). For one-year, three-years, and five-years of survival, the AUC of the relevant ROC curve was 0.582, 0.658, and 0.777, respectively, indicating that the prognostic indicator based on LOX expression showed potential to forecast survival (Fig. 6d). The survival significance of LOX expression was then analyzed using GSE84437, GSE62254, GSE29272, and GSE57303. In the GSE84437 and GSE62254 datasets, survival analyses revealed that patients with high LOX levels had a poor OS (Fig. 6e and f). However, no clear relationship between LOX expression and OS was seen in the GSE29272 or GSE57303 datasets (Fig. 6g and h). To resolve the controversy of results obtained by five datasets, we performed a meta-analysis with 1279 samples from the TGCA and four GEO datasets. As shown in Fig. 6i, the pooled HR and 95 percent CI for the correlation between high LOX expression and OS were 1.24 (1.11–1.37), with no significant inhomogeneity among the five datasets. As a result, our team came to the conclusion that high LOX expression was a robust predictive factor of poor OS among GC patients.

### The association of LOX gene methylation and tumor stage and expression

**Figure 7:**
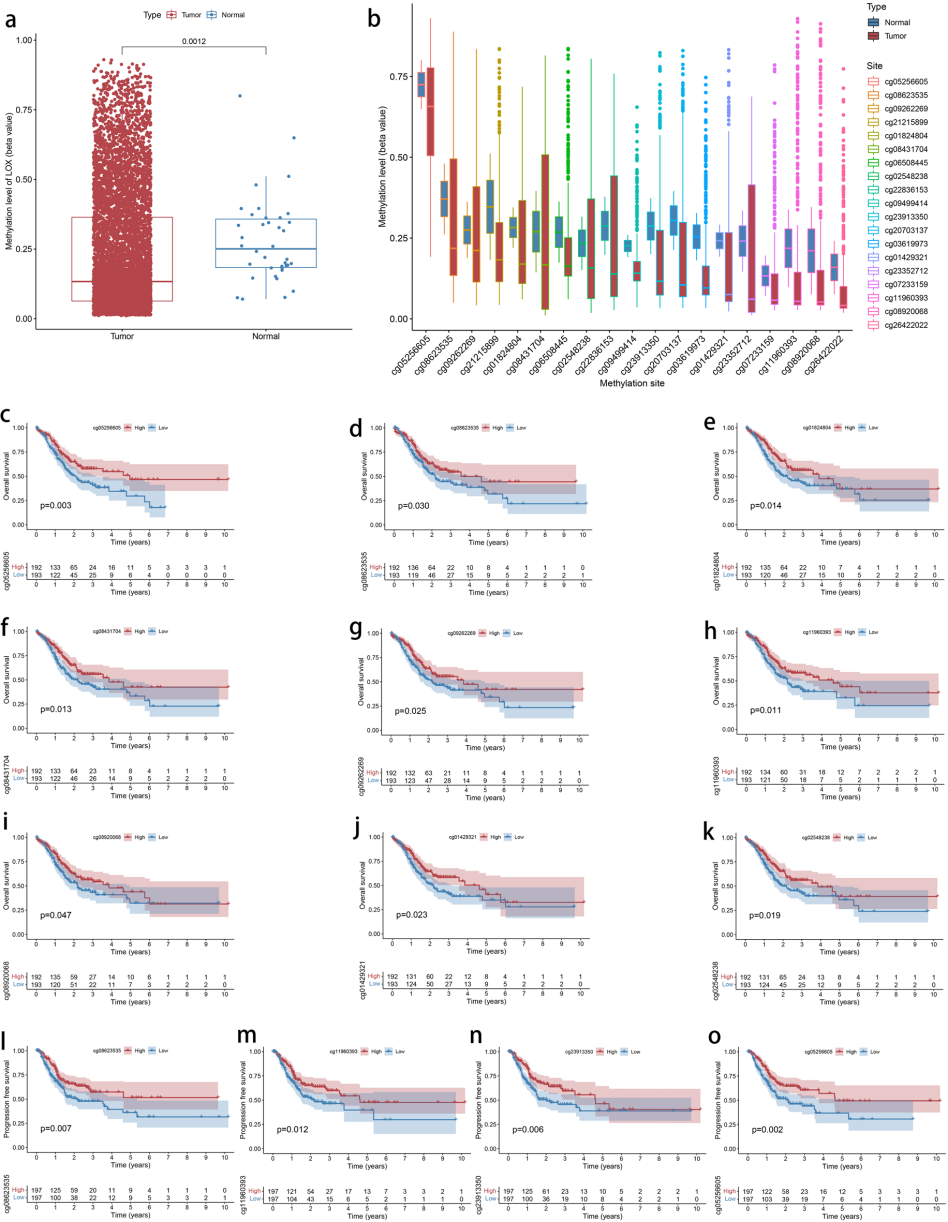
The association of LOX gene methylation and expression with clinicopathologic parameters. (**a**): The LOX gene methylation was expressed at a significantly lower level in GC tissues than in normal. (**b**): The distribution of LOX DNA promoter CpG sites. The low methylation levels of cg sites (cg05256605, cg08623535, cg01824804, cg08431704, cg09262269, cg08920068, cg01429321, cg02548238, and cg11960393) of LOX were related to an unfavorable OS (**c**, **d**, **e**, **f**, **g**, **h**, **i**, **j**, and **k**). The low methylation levels of cg sites (cg08623535, cg11960393, cg23913350, and cg05256605) of LOX were related to an unfavorable PFS (**l**, **m**, **n** and **o**).

A total of 362 samples were obtained from TCGA HumanMethylation450, comprising 360 primary GC and 2 normal samples. We demonstrated the association between LOX gene methylation levels and its location. Our findings imply that the LOX gene is methylationed less in GC tissue than in normal samples (*P* = 0.001) (Fig. 7a).

In addition, there were 19 (cg05256605, cg08623535, cg09262269, cg21215899, cg01824804, cg08431704, cg06508445, cg02548238, cg22836153, cg09499414, cg23913350, cg20703137, cg03619973, cg01429321, cg23352712, cg07233159, cg11960393, cg08920068, and cg26422022) methylation cg sites in the LOX gene. In each cg site, the methylation level of LOX in primary GC is lower than that in para-carcinoma samples (Fig. 7b).

**Figure 8:**
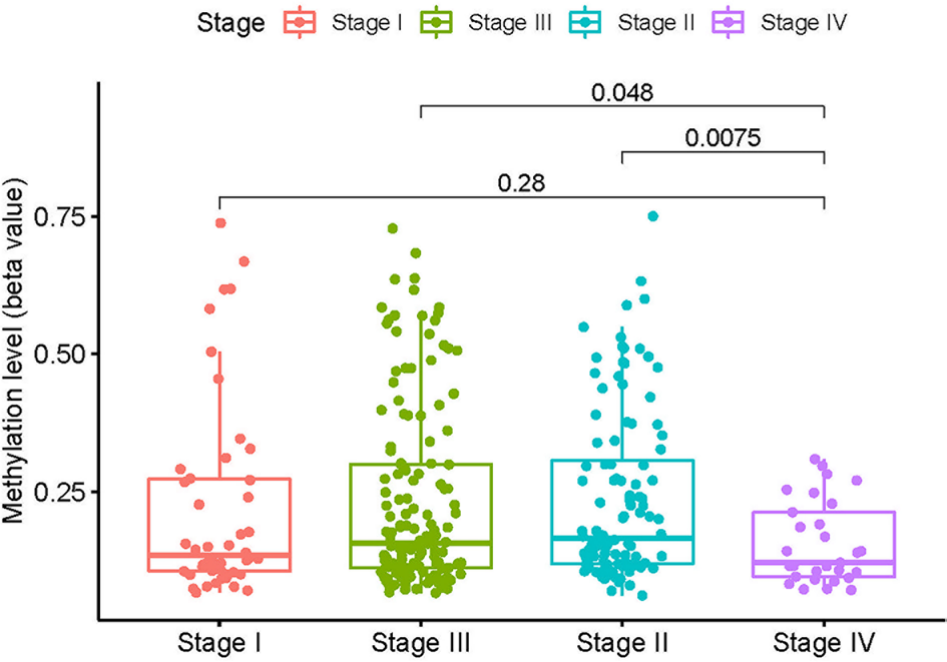
DNA methylation level of LOX is corelated with tumor stage.

Furthermore, the average general LOX gene DNA methylation level and tumor stage were analyzed. Kruskal–Wallis test showed that: DNA methylation level of LOX is corelated with tumor stage (stageII vs stageIV, stageIII vs stage IV) (all *p* < 0.05; Fig. 8).

### Validation of various methylation cg sites in GC for prognostic values

The prognostic significance of methylation cg sites of LOX in GC patients from the TCGA database was investigated using Kaplan–Meier survival analysis. We found that cg sites (cg05256605, cg08623535, cg01824804, cg08431704, cg09262269, cg08920068, cg01429321, cg02548238, and cg11960393) that have low methylation levels of LOX were related to an unfavorable OS (*P* = 0.003, 0.03, 0.014, 0.013, 0.025, 0.047, 0.023, 0.019 and 0.011, respectively) (Fig. 7c–k), whereas other methylation cg sites (cg21215899, cg06508445, cg22836153, cg09499414, cg23913350, cg20703137, cg03619973, cg23352712, cg07233159, and cg26422022) did not have a prognostic value for GC patients (P = 0.604, 0.088, 0.068, 0.584, 0.071, 0.108, 0.287, 0.058, 0.297 and 0.404, respectively) (Supplementary Material 4a-j).

In addition, we found that the low methylation levels of cg sites (cg08623535, cg11960393, cg23913350, and cg05256605) of LOX were related to unfavorable PFS (*P* = 0.007, 0.012, 0.006 and 0.002, respectively) (Fig. 7l–o), whereas other methylation cg sites (cg01429321, cg01824804, cg02548238, cg03619973, cg06508445, cg07233159, cg08431704, cg08920068, cg09262269, cg09499414, cg20703137, cg21215899, cg22836153, cg23352712 and cg26422022) were not related to prognostic value for GC patients (*P* = 0.132, 0.14, 0.06, 0.835, 0.617, 0.157, 0.094, 0.089, 0.103, 0.43, 0.07, 0.396, 0.36, 0.159 and 0.217, respectively) (Supplementary Material 5a-o).

## Discussion

Tumor initiation and growth depend on their microenvironment. Previous study reported that the expression of LOX may have a significant impact in conditioning various TME. LOX might be used as a diagnostic and prognostic biomarker, even a new therapeutic target^30,31^. Currently, an increasing number of studies explore the relationship of LOX expression in GC with clinicopathological parameters and prognosis^19,21,27^. But the results are seriously inconsistent. Mongkolrob R et al*.* have reported the relationship between LOX expression and pan-cancers using meta-analysis^32^. However, only four studies were pooled to discuss the OR for LOX expression in gastrointestinal cancers and gastric cancer was not the main focus. The conflict between LOX expression and clinical parameters and prognosis value still exists in gastric cancer. Our research investigated the relationship in expression and methylation of LOX in GC with its clinicopathological parameters and prognostic value. We pooled seven clinical studies for meta-analysis, including 1435 patients. We also used the TCGA and GEO databases for bioinformatics analysis. Combining our meta-analysis and bioinformatic exploration, we draw the following conclusions: (I) LOX expression was related to lymph node metastasis and tumor distant metastasis of GC patients but not correlated with gender, tumor differentiation, Lauren classification, and tumor depth of invasion. (II) the OS of patients with GC who expressed a high level of LOX was significantly poorer than that of patients who expressed a low level. (III) Low levels of methylation at some cg sites in the LOX gene were significantly related to poor OS and PFS, and methylation levels of LOX are negatively correlated with advanced tumor stage. (IV) LOX is a significant diagnostic and prognosis biomarker in GC.

Earlier studies have indicated that LOX seems to have a crucial role in the metastasis of many malignancies by regulating epithelial mesenchymal transition (EMT) and the establishment of premetastatic niches^27,33–35^. The establishment of a pre-metastatic niche (PMN), which offers a receptive and supportive environment, is a critical step during GC metastasis^36^. Therefore, LOX may play an important role in GC. Many studies suggest an association between LOX expression and clinicopathological parameters in GC. Kasashima et al*.* reported that LOX expression is positively corelated with lymph node metastasis and tumor distant metastasis^27^. However, the relationship between LOX expression and clinicopathological characteristics in GC is controversial. Lai, H. et al. reported that LOX expression is not statistically associated with lymph node metastasis and tumor metastasis^20^. Han YL et al*.* reported that the expression of LOX was positively correlated with the tumor depth of invasion^19^. But the expression of LOX was negatively correlated with tumor depth of invasion, which has also been reported in clinical studies^22^. Our meta-analysis combining seven clinical studies showed that LOX expression was associated with lymph node metastasis and tumor metastasis of GC patients but not correlated with gender, tumor differentiation, Lauren classification, and tumor depth of invasion.

Many researchers have found a relationship between LOX expression and the prognosis of GC. The expression of LOX may enhance many independent risk factors for OS or DFS, including macrophage infiltration, ECM remodeling, angiogenesis, neo-microvessel maturation, MMP-9 secretion, and type IV collagen breakdown^21^. Han et al. reported that LOX may be a poor predictive biomarker that is correlated with a poor prognosis^19^. However, the association between LOX expression and OS in GC patients is still being disputed. Kaneda A et al. reported that LOX is a tumor suppressor gene and correlated with well prognosis^23^. This may be because LOX is an extracellular enzyme that causes collagen and elastin to cross-link. Loss of this function might facilitate the spread of tumor GC cells^37^. Another possible reason may be that the LOX propeptide exerts a tumor suppressor effect^38^. Our meta-analysis revealed that compared to GC patients with low LOX expression, the OS was significantly poorer with high LOX expression, regardless of whether the data came from clinical studies or bioinformatic datasets.

Abnormal DNA methylation has been shown to alter the tumor microenvironment and may be used to diagnose disease and predict prognosis. The methylation profile is critical for identifying the functional state of genes since their expression is reliant on the methylation status of the DNA CpG island^39,40^. According to research, abnormal DNA methylation of certain genes may have a key role in GC pathophysiology and development^41^. Oue, Naohide et al. also reported that the level of promoter DNA methylation in LOX is strongly negative associated with LOX expression^42^. Previous studies reported that LOX methylation may corelated with poor prognosis^23,23^. But it is not consistent with the conclusion of this study. So we further explore the association between methylation levels of cg sites and prognosis. We found that LOX expression is higher but methylation is lower in GC compared with healthy controls. We also found that the low methylation levels of cg sites (cg05256605, cg08623535, cg01824804, cg08431704, cg09262269, cg08920068, cg01429321, cg02548238 and cg11960393) of LOX were significantly related to a poor OS for GC patients. The low methylation levels of cg sites (cg08623535, cg11960393, cg23913350, and cg05256605) of LOX were related to poor PFS. These findings demonstrate that methylation cg sites might be used as prognostic and therapeutic targets for GC. In addition, we found that methylation levels of LOX are negatively correlated with advanced tumor stage. These findings are very consistent with our elsewhere results within this present study and strongly supported that LOX expression was correlated with poor prognosis.

Several studies have reported the diagnostic and prognostic value of LOX in GC patients. Zhu J et al*.* reported that overexpression of LOX may serve as a biomarker for a poor prognosis in GC^30^. Lai H et al. reported that combining LOX with CEA, and CA199 might increase the sensitivity of lymph node and peritoneal metastasis diagnosis in GC patients^20^. There is growing evidence that DNA methylation analysis is critical for early illness detection, therapy effectiveness evaluation, and prognosis estimation in GC patients^43^. Our integrated analysis of DNA methylation and gene expression supports LOX as a significant diagnostic and prognosis biomarker in GC.

Our study also has some potential limitations. Firstly, despite our comprehensive literature search strategy, the sample size from clinical studies was not enough to perform the clinical parameter analysis of any subgroup of GC patients. The results of publication bias may not have had a significant impact due to the studies that were enrolled in less than nine. Secondly, all of the clinical studies were from east Asia, which may have caused heterogeneity. In addition, our results are not definitive, but they will contribute in establishing a hypothesis or a research strategy for further studies. Furthermore, the classification criteria (high/low) for IHC of LOX expression in GC samples in seven clinical studies were not totally the same. Thus, further studies with larger sample sizes are needed to confirm our findings.

## Conclusion

We performed a meta-analysis to clarify the association and existing conflict in LOX expression with clinicopathological parameters and prognostic value by using clinical studies. We also perform bioinformatic analysis using TCGA and GEO databases to further demonstrate and explore the function of LOX. To sum up, our work found that LOX expression was related to lymph node metastases and tumor metastasis of GC patients but not related to gender, tumor differentiation, Lauren classification, and tumor depth of invasion. In addition, compared to GC patients with low LOX expression, the OS was significantly poorer with high LOX expression. Furthermore, low levels of methylation at some cg sites in the LOX gene were found to be strongly correlated with poor OS and PFS and methylation levels of LOX are negatively correlated with advanced tumor stage. Integrative methylation and gene expression analysis support LOX in TME as a specific diagnostic and prognosis biomarker in GC.

## Supplementary Information

Supplementary Information 1.

Supplementary Information 2.

Supplementary Information 3.

Supplementary Information 4.

Supplementary Information 5.

## Data Availability

The meta-analysis data generated or analyzed during this study is included in this published article and its supplementary material files. The datasets generated and analysised during the current study are available in the GEO and UCSC Xena repository, https://www.ncbi.nlm.nih.gov/geo/ and https://xena.ucsc.edu/.
