# Supplementary material for: Clinicopathological and prognostic value of lysyl oxidase expression in gastric cancer: a systematic review, meta-analysis and bioinformatic analysis
Source: Sci Rep. 2022 Oct 6;12:16786. doi: 10.1038/s41598-022-21402-1 (PMC9537423; doi:10.1038/s41598-022-21402-1)
Supplement: Supplementary file 2 — Supplementary Information 2. [file 41598_2022_21402_MOESM2_ESM.docx]

Supplementary Material 2: Other characteristics of the eligible studies

| No. | First author | Differentiation  (Low and middle/High) | Depth of invasion  (T3+T4/T1+T2) | Lymph node metastasis  (Metastasis/None- metastasis) | Tumor metastasis  (M1/M0) | Antibody |
| --- | --- | --- | --- | --- | --- | --- |
| 1 | Han YL (19) | 107/33 | 122/18 | 106/34 | 81/59 | Abcam |
| 2 | Peng C (21) | - | 157/27 | 92/92 | 7/177 | Abcam [ab31328] |
| 3 | Kasashima H (27) | - | - | 285/256 | 98/446 | Novus Biologicals |
| 4 | Lai H (20) | 96/109 | 148/67 | 90/125 | 72/143 | Abcam |
| 5 | Yalin Han (28) | 125/41 | 146/20 | 66/100 | 20/146 | Abcam [ab312238] |
| 6 | Zhang Q (29) | 110/51 | - | 68/93 | - | Abcam |
| 7 | He Jie (22) | - | 17/8 | - | - | Unclear |
